# Change of Direction Deficit in National Team Rugby Union Players: Is There an Influence of Playing Position?

**DOI:** 10.3390/sports7010002

**Published:** 2018-12-21

**Authors:** Tomás T. Freitas, Pedro E. Alcaraz, Chris Bishop, Julio Calleja-González, Ademir F. S. Arruda, Aristide Guerriero, Valter P. Reis, Lucas A. Pereira, Irineu Loturco

**Affiliations:** 1 Research Center for High Performance Sport, Catholic University of Murcia, 30107 Murcia, Spain; tfreitas@ucam.edu (T.T.F.); palcaraz@ucam.edu (P.E.A.); 2 Faculty of Sport Sciences, Catholic University of Murcia, 30107 Murcia, Spain; 3 Faculty of Science and Technology, London Sports Institute, Middlesex University, London NW4 1RL, UK; C.Bishop@mdx.ac.uk; 4 Laboratory of Sport Performance Analysis, Faculty of Physical Education and Sport Sciences, University of Basque Country, 01006 Vitoria, Spain; julio.calleja.gonzalez@gmail.com; 5 Brazilian Rugby Confederation, São Paulo 01407911, Brazil; ademirf.schultz@gmail.com (A.F.S.A.); guerrieroaristide1@gmail.com (A.G.); 6 Department of Motor, Human and Health Sciences, University of Rome Foro Italico, 00135 Rome, Italy; 7 NAR – Nucleus of High Performance in Sport, São Paulo 04753060, Brazil; valterpreis@gmail.com (V.P.R.); lucasa_pereira@outlook.com (L.A.P.)

**Keywords:** team sports, agility, athletes, sprint velocity, muscle power

## Abstract

The aim of this study was to investigate the change of direction (COD) ability and deficits of elite rugby union players, discriminating between position (backs and forwards), and between “faster and slower players”, in multiple COD tasks. Twenty-four male rugby union players from the Brazilian senior National team completed the following assessments: Squat and countermovement jumps; drop jump; standing long jump, horizontal triple jumps; 40-m linear sprint; Pro-agility, L-Drill, and Zig-zag COD tests; and squat 1-repetition maximum. The differences between backs and forwards and between faster and slower performers were examined using magnitude-based inferences. Backs were faster (in both linear and COD speed tests) and jumped higher than forwards. Moreover, they generated an inferior sprint momentum. No differences were found in COD deficit between playing positions. However, when dividing the sample by median split, faster players outperformed their slower counterparts in all power–speed variables and presented higher COD deficits. These results suggest that separating rugby players by playing position might not discriminate players with different COD skills and that the median split analysis is more sensitive to identifying these differences. Furthermore, the present data indicate that faster rugby players are less efficient at changing direction and tolerating higher approach velocities in COD maneuvers.

## 1. Introduction

Rugby union is a team sport characterized by repeated high-intensity efforts, including noncontact activities (e.g., sprinting, jumping, and changing direction) and player-to-player contact actions (e.g., tackling, scrummaging, and ruck contests) [1]. The particular demands of the competition require elite rugby union players to possess substantial levels of strength and power capabilities, and also the ability to achieve high acceleration rates and high speeds over a wide range of distances (i.e., from 10 to 50 m) [1,2]. According to in-match roles, rugby players are commonly grouped into two main positions: Forwards and backs [2]. Since they are frequently required to perform static high-intensity actions (e.g., rucking and mauling), forwards are significantly heavier, taller, and stronger [2,3]. On the other hand, backs usually cover greater distances and execute more sprints during competitions [4], thus tending to be lighter, faster, and more powerful than forwards [2,3].

Despite the differences reported between forwards and backs using time–motion analysis, both positions are required to perform multiple (and successive) accelerations, decelerations, and changes of direction (COD) during a match [1,4]. For these reasons, several studies have focused on identifying the main determinants of COD ability and maneuverability (i.e., ability to maintain velocity in a curvilinear running pattern [5]) in this team sport [6,7,8]. For example, Delaney et al. [7] investigated the relationships between COD performance and several anthropometric and speed–power outcomes in professional rugby players. The authors concluded that relative strength and power were the variables with greater influence on COD ability; nonetheless, players were not analyzed separately by position. Conversely, Gabbett et al. [9] examined the differences in COD speed between positions, observing that “props” (considered forwards) were significantly slower to change direction than all other positions (i.e., “backrowers” and “outside backs”; considered backs). Nevertheless, the sample in that study was composed of sub-elite rugby players. Thus, the necessity to better understand and define the mechanisms involved in specific COD performance of elite forwards and backs still persists. Unquestionably, more in-depth knowledge of the specific requirements of each position would help coaches and sport scientists to develop more effective and tailored COD-related training strategies.

More recently, the “COD deficit” has been proposed as an adequate method for assessing COD ability in different team sports, such as netball [10,11], soccer [12], handball [13], and youth cricket and basketball [14]. The COD deficit reports the additional time required to perform a directional change when compared to the time needed to cover the same distance in a linear sprint [11] or, alternatively, the difference in velocity between the linear sprint and a COD task of equal distance [13]. In summary, this novel variable is an indicator of the athlete’s efficiency in changing direction, based on his or her maximum linear velocity, which provides a more precise measurement of COD ability as a separate quality [11,12]. Curiously, some studies performed with elite soccer [12] and handball players [13] reported that faster and more powerful athletes tend to be less efficient when changing direction (i.e., presenting greater COD deficits). For instance, Pereira et al. [13] observed that Olympic male handball players outperformed their female counterparts in a wide variety of speed and power tests as well as presenting greater COD deficits in 100° directional change maneuvers.

Apart from the abovementioned differences between player positions, Nakamura et al. [3] reported that elite forwards (heavier and stronger than backs) generated greater sprint momentum (i.e., a product of body mass and sprint velocity), hence, inertia. This aspect is important to consider, given that higher momentum is closely associated with greater braking and propulsive forces (in successive decelerations–accelerations) and longer ground contact times during multidirectional maneuvers [15], which certainly affects COD performance. In addition, differences between positions should be accounted for because specific factors, such as maximum straight speed, anthropometric characteristics, and leg muscle qualities, vary greatly between positional roles [2,3], possibly influencing the efficiency to change direction [16]. Nevertheless, no previous study has separately assessed COD ability using the COD deficit in rugby union players according to their playing positions. To date, there is no evidence as to whether backs, by being faster and more powerful, present higher COD deficits or, on the contrary, forwards are less efficient at changing direction by being heavier and possessing higher sprint momentum. Therefore, the aim of this study was to investigate in depth, through a more comprehensive statistical approach (also using the median split analysis), the COD ability and deficits of National team rugby union players, discriminating between backs and forwards, and between “faster and slower players”, in multiple COD and maneuverability tests.

## 2. Materials and Methods

### 2.1. Experimental Design

This cross-sectional comparative study aimed to assess the COD deficit in different COD and maneuverability tasks in distinct playing positions (i.e., backs and forwards) of National team rugby union players and in faster and slower performers. Athletes completed all physical assessments on the same day, in the following order: Squat and countermovement jumps (SJ and CMJ); 45-cm height drop jump (DJ45); standing long jump (SLJ) and horizontal triple jumps (HTJ); 40-m linear sprint; Pro-agility, “L” (L-drill), and Zig-zag COD tests; and 1-repetition maximum test (1-RM) in the squat exercise. All players were familiarized with the testing procedures due to constant training and assessments at the same sports facilities. Prior to data collection, athletes performed a standardized warm-up protocol that included general (i.e., running at a moderate pace for 10 min followed by active lower limb stretching for 3 min) and specific exercises (i.e., submaximal attempts at each test).

### 2.2. Participants

Twenty-four elite male rugby union players (backs: n = 13; age: 22.0 ± 2.3 years; body mass: 84.0 ± 7.9 kg; height: 179.2 ± 7.3 cm; forwards: n = 11; age: 23.1 ± 3.2 years; body mass: 108.3 ± 12.8 kg; height: 185.6 ± 8.8 cm) from the Brazilian senior National team (overall champion in the most recent edition of the South-America Rugby Championship) participated in the study. Rugby players were tested in the final phase of preparation for important national and international competitions. The study was approved by the local Ethics Committee (926.260) and all participants were informed of the inherent risks and benefits associated with study participation before signing informed consent forms.

### 2.3. Vertical Jumps

Vertical jump ability was assessed using the SJ, CMJ, and DJ45. In the SJ, players were instructed to maintain a static position with a 90° knee flexion angle for 2 s before performing a jump attempt without any preparatory movement. In the CMJ, players performed a downward movement followed by a complete explosive extension of the lower limbs. The depth of the countermovement was self-determined to avoid changes in the jumping coordination pattern. In the DJ45, participants stepped off the box with knees and ankles fully extended and were instructed to touch the ground in a similarly extended position and to jump “as high and as fast as possible” to ensure the validity of the test [17]. All jumps were executed with the hands on the hips. Five attempts at each jump were performed interspersed by 15-s intervals. CMJ and SJ heights were determined based on flight time using a previously validated contact mat (Elite Jump^®^, S2 Sports, São Paulo, Brazil) [18]. The highest SJ and CMJ were used for data analysis. For the DJ45, the best reactive strength index (RSI) was taken from the jump height divided by the ground contact time before the take-off.

### 2.4. Horizontal Jumps

Horizontal jump ability was evaluated with the SLJ and HTJ. Athletes started from a standing position, with knees bent, and arm swing was allowed to provide maximal forward drive. In the SLJ, players jumped as far as possible using both legs. For the HTJ, athletes performed three maximal horizontal bilateral jumps in sequence. The jump distance was determined using a metric tape measure (Lufkin, L716MAGCME, Appex Tool Group, Sparks, MD, USA), from the take-off line to the nearest point of landing contact (i.e., back of the heels). Three attempts at each jump were allowed, interspersed by 30-s intervals, and the trial with the longest distance was retained for the analyses.

### 2.5. Linear Sprint Velocity

Linear sprint testing was conducted on an indoor running track. Five pairs of photocells (Smart Speed, Fusion Equipment, Brisbane, Australia) were positioned at the starting line and at the distances of 10, 20, 30, and 40 m along the sprinting course. Athletes positioned themselves 0.3 m behind the starting line and performed two maximal sprints, from a standing position. A 3-min rest interval was allowed between trials and the fastest time was considered for analysis. Sprint momentum (kg·m·s^−1^) was obtained by multiplying the athlete’s body mass by the respective velocity during the linear sprint.

### 2.6. Change of Direction Speed and Maneuverability

**Figure 1:**
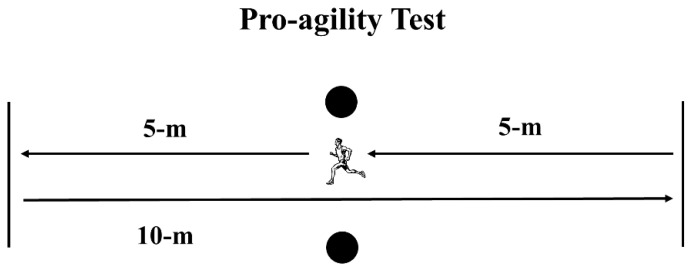
Schematic presentation of the Pro-agility test. Circles represent the position of the photocells.

Players performed three COD tasks: The Pro-agility, L-drill, and Zig-zag tests. For all tests, the trials were separated by a 5-min resting interval. In the Pro-agility test (5–10–5), participants started in a standing position over the starting line, facing one of the photocells. At the instructor’s signal, athletes turned and sprinted 5 m, touching the line with hand, then turned 180° and ran 10 m to touch the other line. Finally, they sprinted 5 m towards the finishing line, covering a total distance of 20 m (Figure 1). Athletes performed two attempts starting to the right side and two to the left. The fastest time of the four attempts was considered for analysis. 

**Figure 2:**
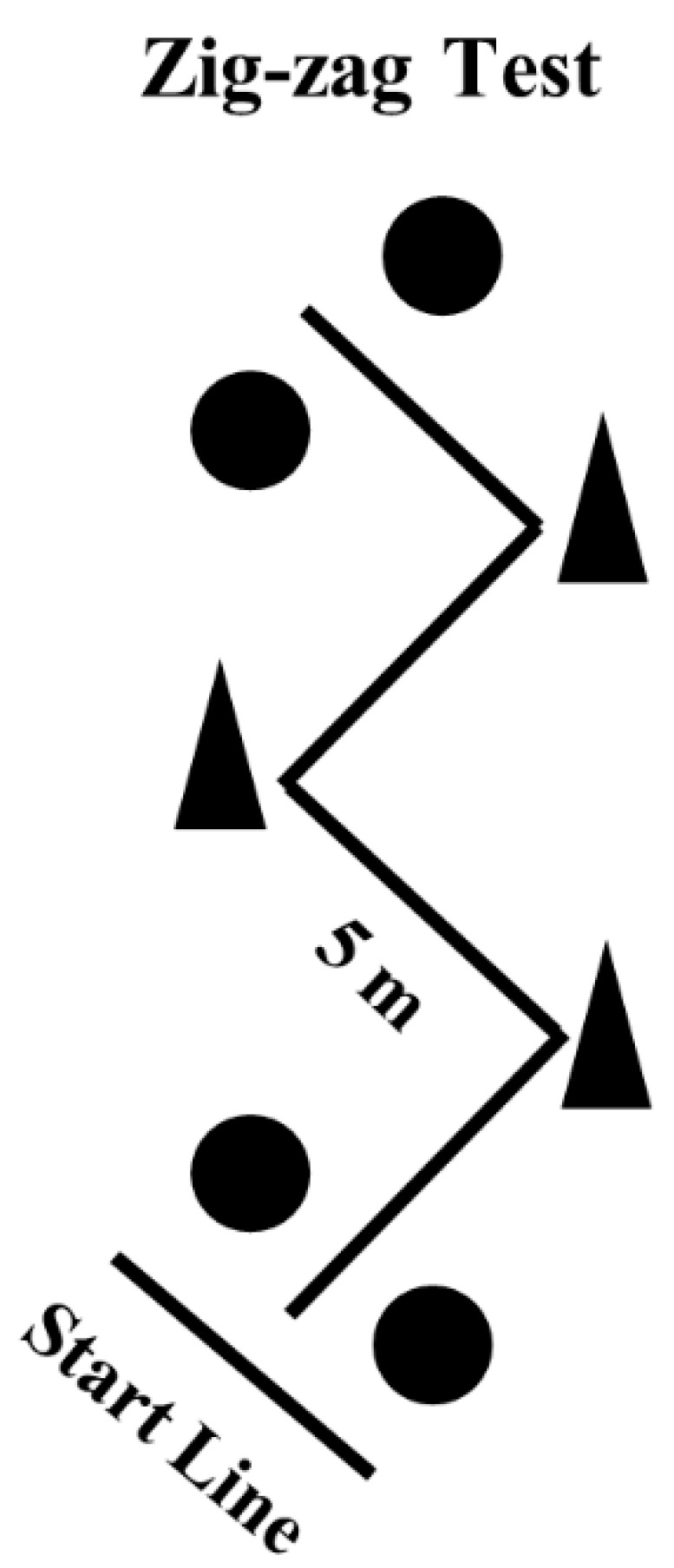
Schematic presentation of the Zig-zag test. Circles represent the position of the photocells.

The Zig-zag test is considered a maneuverability test that comprised four 5-m sections (total 20 m linear distance) marked with cones set at 100° angles (Figure 2), requiring the athletes to decelerate and accelerate as fast as possible around each cone. Starting from a standing position with the front foot placed 0.3 m behind the first pair of timing gates (Smart Speed, Fusion Equipment, Brisbane, Australia) (i.e., starting line), athletes were encouraged to complete the test as quickly as possible by crossing the second pair of timing gates, placed over the finishing line. The fastest time from the two trials was retained for further analysis.

**Figure 3:**
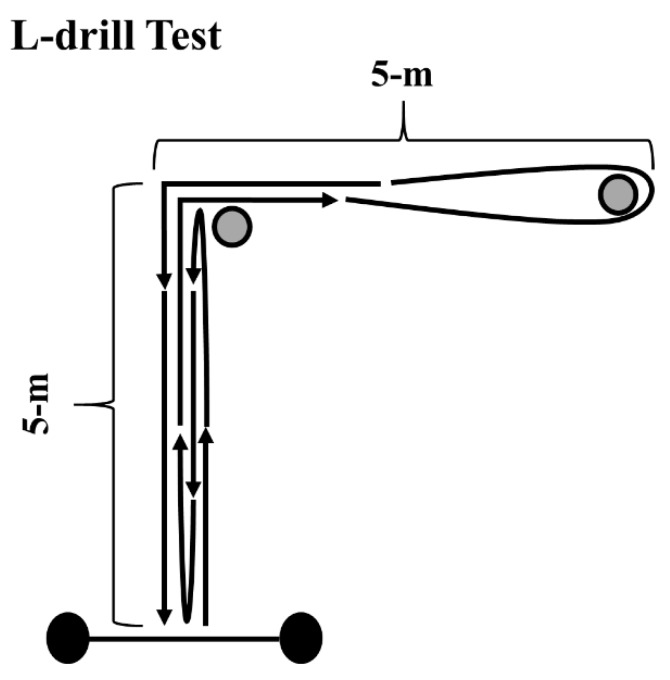
Schematic presentation of the L-drill test. Black circles represent the position of the photocells and grey circles the position of the cones.

In the L-drill, considered a COD speed and maneuverability test, subjects started in a standing position, and were required to sprint forward 5 m, touch the line, and sprint back to the starting line. Next, they performed a 180° directional change and sprinted to the first cone, cut 90°, and circled the second cone. Lastly, players performed a final 90° cut before completing the 30-m test by sprinting through the photocells placed on the finishing line (Figure 3). Athletes performed two attempts starting to the right side and two to the left. The fastest time of the four attempts was considered for analysis.

To evaluate COD and maneuverability as separate qualities (isolating the acceleration capability of the athlete), an adapted COD deficit calculation was used, as described elsewhere [11,12]. Each COD deficit was calculated based on the difference between linear sprint and COD velocities of tests of equal distance, as follows: (I) Pro-agility: 20-m velocity − Pro-agility test velocity; (II) Zig-zag: 20-m velocity − Zig-zag test velocity; and (III) L-drill: 30-m velocity − L-drill velocity [11,12].

### 2.7. One-Repetition Maximum Test in the Squat Exercise

The 1RM test was performed using an Olympic barbell. The testing protocol was adapted from the procedures proposed by Brown and Weir [19]. Prior to the test, athletes performed 3 specific squat warm-up sets. In the first set, participants performed 4 repetitions with 50% of the estimated 1RM (i.e., based on prior assessments); in the second set, they performed 3 repetitions with 60% of the estimated 1RM, and in the third set, they performed 2 repetitions with 70% of the estimated 1RM. A 3-min resting interval was allowed between sets. Three minutes after the warm-up, participants performed up to 5 attempts (≈80%, 90%, 95%, and (1–2 repetitions) >95% of the estimated 1RM) to obtain the 1RM load (e.g., maximum weight that could be lifted once using the proper technique), with a 3-min interval between attempts. To account for the differences in players’ body mass, values were normalized by dividing the 1RM load by the athletes’ body mass.

### 2.8. Statistical Analysis

Data are presented as means ± standard deviations (SD). The statistical analysis was performed with the SPSS^®^ software package version 22.0 for Windows (SPSS, Inc., Chicago, IL, USA) and data normality was confirmed using the Shapiro–Wilk test. A median split analysis was used to divide rugby players into two groups according to their linear sprint velocity (faster and slower players). Differences in all performance outcomes between backs and forwards and between the faster and slower players were analyzed using magnitude-based inferences [20]. The quantitative chances of finding differences between the variables tested were assessed qualitatively as follows: <1%, *almost certainly not*; 1% to 5%, *very unlikely*; 5% to 25%, *unlikely*; 25% to 75%, *possibly*; 75% to 95%, *likely*; 95% to 99%, *very likely*; >99%, *almost certainly*. If the chances of having better and poorer results were both >5%, the true difference was deemed *unclear* [20]. The magnitudes of the differences for the comparisons in all variables were analyzed using the Cohen’s *d* effect size (ES) [21,22]. The magnitudes of the ES were qualitatively interpreted using the following thresholds: <0.2, trivial; ≥0.2, small; ≥0.6, moderate; ≥1.2, large; ≥2.0, very large; and ≥4.0, almost perfect [22]. Intraclass correlation coefficients were calculated for the tests performed in this study and were all >0.90.

## 3. Results

Forwards were found to be *likely* taller (ES = 0.79) and *almost certainly* heavier (ES = 2.27) than backs, while the age comparison between playing positions was rated as *unclear* (ES = 0.39). In addition, considering the median split analysis, faster players were found to be *very likely* lighter than the slower ones (faster: 88.5 ± 9.6 kg; slower: 104.2 ± 18.6 kg; ES = 1.00), while no differences were observed for age (faster: 22.8 ± 2.9 years; slower: 23.3 ± 1.7 years; ES = 0.23) or height (faster: 181.7 ± 7.6 cm; slower: 185.0 ± 10.6 cm; ES = 0.35) when comparing faster and slower players.

**Figure 4:**
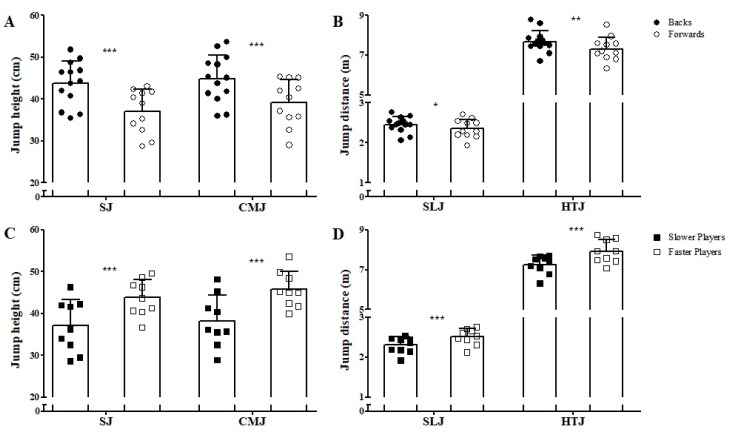
Comparisons of the vertical (**A**,**C**) and horizontal jumps (**B**,**D**) between backs and forwards and between faster and slower groups. The figure demonstrates the mean and standard deviation bars along with individual data points to show the full spread of data. SJ: Squat jump; CMJ: Countermovement jump; SLJ: Standing long jump; HTJ: Horizontal triple jump; * *possibly* different; ** *likely* different; *** *very likely* different.

Figure 4 shows the vertical and horizontal jump comparisons between backs and forwards and between faster and slower athletes. Vertical jump performance was *very likely* higher in the backs, when compared to forwards (ES = 1.25 and 1.00 for SJ and CMJ, respectively) and *very likely* higher in the faster players, in comparison to the slower (ES = 1.19 and 1.36 for SJ and CMJ, respectively). DJ45 RSI was *likely* higher in the backs in comparison to forwards (backs: 1.35 ± 0.52 mm·ms^−1^; forwards: 0.99 ± 0.23 mm·ms^−1^; ES = 0.84). In addition, the faster players demonstrated a *likely* higher DJ45 RSI than the slower group (faster players: 1.24 ± 0.24 mm·ms^−1^; slower players: 1.01 ± 0.29 mm·ms^−1^; ES = 0.83). Regarding horizontal jump ability, backs demonstrated *possibly* and *likely* better performances than forwards in the SLJ (ES = 0.45) and HTJ (ES = 0.63), respectively. Meanwhile, the faster athletes demonstrated *very likely* higher performances in the horizontal jumps than the slower players (ES = 1.02 and 1.26 for SLJ and HTJ, respectively).

**Figure 5:**
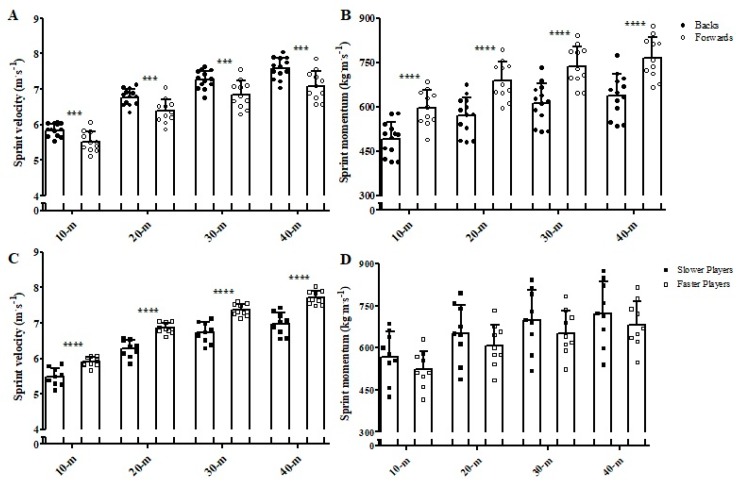
Comparisons of the sprint velocity (**A**,**C**) and momentum (**B**,**D**) in the different distances tested between backs and forwards and between faster and slower groups. The figure demonstrates the mean and standard deviation bars along with individual data points to show the full spread of data. *** *very likely* different; **** *almost certainly* different.

Regarding linear sprint velocity (Figure 5), backs achieved *very likely* higher velocities than forwards in all distances tested (ES = 1.37, 1.31, 1.26, and 1.33, for sprint velocity in 10, 20, 30, and 40 m, respectively). *Almost certainly* higher sprint momentum (Figure 5) was observed for forwards in comparison to backs in all distances (ES = 1.76, 1.84, 1.86, and 1.77, for sprint momentum in 10, 20, 30, and 40 m, respectively). The faster players showed *almost certainly higher* sprint velocities in all distances tested when compared to the slower ones (ES = 1.93, 2.65, 2.55, and 1.63, for sprint velocity in 10, 20, 30, and 40 m, respectively). The comparisons of the sprint momentum between faster and slower players were all rated as *unclear* (ES = 0.58, 0.48, 0.45, and 0.40, for sprint momentum in 10, 20, 30, and 40 m, respectively).

**Figure 6:**
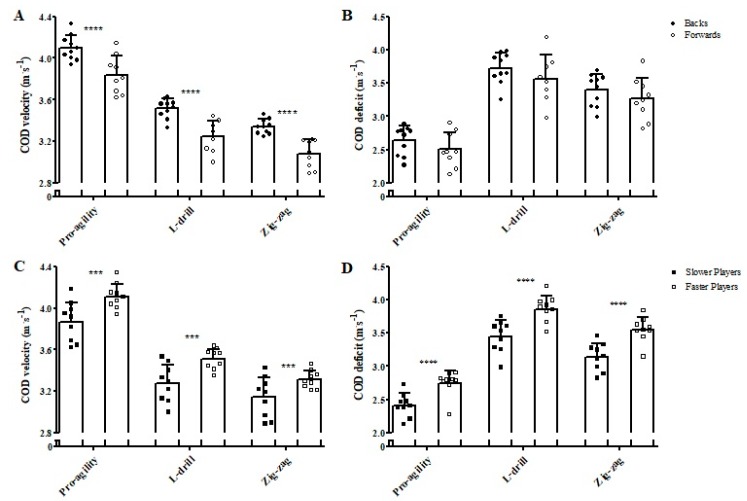
Comparisons of the change of direction (COD) velocity (**A**,**C**) and COD deficit (**B**,**D**) in the three different tests performed between backs and forwards and between faster and slower groups. The figure demonstrates the mean and standard deviation bars along with individual data points to show the full spread of data. *** *very likely* different; **** *almost certainly* different.

Figure 6 shows the comparisons of the COD velocity and COD deficit between backs and forwards and between faster and slower athletes in the three different tests performed. Backs showed *almost certainly* higher COD velocities in all tasks when compared to forwards (ES = 1.67, 2.12, and 2.40 for Pro-agility, L-Drill, and Zig-zag, respectively). No meaningful differences were observed for any COD deficits calculated when comparing backs and forwards (ES = 0.55, 0.52, and 0.47, for Pro-Agility, L-Drill, and Zig-zag, respectively). Faster athletes demonstrated *very likely* higher COD speeds in all tests in comparison to the slower players (ES = 1.42, 1.52, and 1.05, for Pro-Agility, L-Drill, and Zig-zag, respectively). In addition, the faster group showed *almost certainly* higher COD deficits in all tests when compared to the slower group (ES = 1.80, 1.83, and 1.98, for Pro-Agility, L-Drill, and Zig-zag, respectively).

Concerning maximum strength, backs showed *almost certainly* higher 1RM, relative to body mass, in comparison with forwards (backs: 1.84 ± 0.26 kg·kg^−1^; forwards: 1.45 ± 0.20 kg·kg^−1^; ES = 1.62). Meanwhile, faster athletes demonstrated a *likely* higher relative 1RM when compared to the slower players (faster players: 1.84 ± 0.20 kg·kg^−1^; slower players: 1.50 ± 0.37 kg·kg^−1^; ES = 1.16). 

## 4. Discussion

The main purpose of the present research was to assess COD ability and deficits of National team rugby union players discriminating between playing position (i.e., backs and forwards) and linear sprint velocity (using a median split analysis to separate faster and slower athletes). In line with previous research [2,3,23], backs were found to: (I) Be lighter, (II) be faster (both in linear sprinting and COD and maneuverability tests), and (III) jump higher than forwards. Moreover, they generated an inferior sprint momentum. No differences were found in COD or maneuverability efficiency when players were discriminated by position; however, remarkably, when dividing the sample by median split, the faster players outperformed their slower peers in all power–speed variables and presented higher COD deficits.

As expected, forwards generated greater sprint momentum than backs, in accordance with previous literature [3,24,25]. In collision sports such as rugby, sprint momentum is of considerable importance [26]. It has been suggested that heavier players who achieve high velocities are able to crash against opposing players with greater momentum, hence driving defenders backward and providing their team with an attacking advantage [26]. Thus, it is crucial for coaches and sports scientists to develop training strategies focusing on improving this aspect. Interestingly, when assessing sprint momentum using the median split analysis, no differences were attained between faster and slower players, despite the latter group displaying a *very likely* higher body mass (15% difference). From a mechanical point of view, this indicates that the higher velocities achieved by the faster players counterbalanced their lower body mass [24]. It is important to note that mass positively affects momentum [24,27] but negatively affects velocity, which suggests that an “ideal relationship” between these variables should be obtained to maximize sprint momentum [24]. Taking the above into account, relevant practical applications can be drawn from the present results. Specifically, if coaches want to effectively improve sprint momentum, especially for heavier and slower rugby players (i.e., forwards), training should be directed towards speed development over shorter distances (i.e., ≤20 m) [1], instead of solely focusing on increasing players’ mass. On the other hand, due to their specific match demands, backs must be oriented to develop physical and technical qualities more related to higher speeds (and longer distances, ≤50 m) [1]. As shown in the present study, greater body mass does not necessarily imply higher sprint momentum in elite level rugby players if they are unable to achieve high sprint velocities.

Regarding COD ability and maneuverability, backs and faster players presented greater velocities in all performed tests (i.e., Pro-agility, L-drill, and Zig-zag). Notably, no differences were found in COD deficit when comparing backs and forwards; by contrast, when using the median split analysis, faster players presented an inferior ability to change direction (i.e., greater COD deficits), independently of the angles of direction changes. This interesting finding indicates that separating rugby players by positional group might not be sensitive enough to discriminate between players of different COD skills, contrary to what has been reported for other speed–power variables (e.g., CMJ, SJ, linear sprint, absolute and relative strength) [2,3,23,25]. Therefore, rugby practitioners are strongly encouraged to assess COD ability and maneuverability using the COD deficit [10,11,12] and, subsequently, apply a median split analysis relative to linear speed to better identify player-specific training needs. This is seemingly a more suitable approach than separating athletes by their positional roles, as it provides a more comprehensive view of their COD performance, allowing coaches to create more effective and tailored (“movement-specific”) training strategies. Given the previously reported importance of COD performance in rugby union [1,4], this appears like a logical suggestion for practitioners.

In fact, when discriminating players based on their linear sprint velocity, faster players presented greater COD deficits than their slower peers in all COD maneuvers, supporting the findings of Loturco et al. [12] and Pereira et al. [13] with elite soccer and handball players, respectively. This tendency seems to be common for different team-sport modalities (e.g., rugby union, soccer, handball), suggesting that the training strategies currently used with professional athletes are preparing them to be faster in straight sprinting actions, but not necessarily to change direction. Furthermore, this indicates that faster athletes are not capable of handling the high approach velocities in a COD maneuver [12]. Therefore, alternative and more multifaceted training approaches less focused on developing linear sprint-related capabilities should be considered, especially if the main goal is to improve the ability to efficiently execute different COD tasks [15]. In this regard, Loturco et al. [12] suggested that drills mimicking game-specific COD maneuvers, emphasizing acceleration–deceleration actions, can potentially decrease the COD deficit in faster and more powerful athletes. In addition, a compelling body of evidence [15,28,29,30,31] indicates that eccentric strength may play an important role in COD ability, namely the capacity to decelerate and tolerate higher breaking forces. For example, a study by de Hoyo and colleagues [30] investigated the effects of a 10-week training intervention with an eccentric overload in soccer players and reported meaningful increases in relative peak braking and propulsive forces and impulses during a side-step and a crossover cutting task. In summary, from a practical perspective, training programs focused on increasing eccentric and reactive strength [29] and COD mechanics may improve the ability to perform directional changes in multiple planes [15] and coaches are advised to include them in their training routines.

The main limitation of the present study is its cross-sectional nature, as it does not allow confirmation of causal relationships between the variables or investigation of the influence of training programs focused on acceleration–deceleration drills or eccentric strength on COD and maneuverability efficiency of professional rugby union players. In addition, due to the elite level and particular characteristics of the sample studied herein, the results obtained are difficult to generalize to other competitive levels or team sports. Future research should focus on identifying whether longitudinal COD and maneuverability training schemes prescribed based on the individual needs of players, assessed through median split analysis, prove to be more efficient in increasing COD capabilities in faster and more powerful athletes (i.e., reducing COD deficits). Moreover, it would be of interest to investigate the effectiveness of training programs that include eccentric exercises, acceleration–deceleration drills, and tailored COD technique-oriented tasks in reducing COD deficits in team-sport athletes. 

## 5. Conclusions 

The results from the present research show that backs outperformed forwards in all speed–power and COD velocity outcomes, but no differences between positions were found in COD deficits. However, when using a median split analysis dividing the sample according to linear sprint velocities, faster players were found to have an inferior COD ability and maneuverability (i.e., higher COD deficits in the Pro-agility, L-drill, and Zig-zag tests). This suggests that separating elite rugby union players by positional group might not be sensitive enough to discriminate between players of different COD skills. Furthermore, it indicates that faster rugby players are less efficient at changing direction and tolerating higher approach velocities in COD maneuvers. Sprint momentum was greater in forwards when compared to backs, but no differences were identified between faster and slower players, implying that the high velocities achieved by the faster athletes compensated for their lower body mass. 

From an applied perspective, coaches are advised to use a median split analysis to more accurately identify rugby players with an inferior COD ability and maneuverability instead of separating players according to their in-match roles. This will enable the prescription of more effective and individualized training programs to improve the athletes’ capability to perform directional changes. The fact that faster players displayed higher COD deficits in all maneuvers suggests that the training strategies currently employed in rugby overemphasize linear sprint and power development in detriment of COD skills. In this regard, if the main goal is to improve the ability to efficiently change direction in multiple planes of movement, alternative and more multifaceted training approaches focused on eccentric overload, reactive strength, and movement-specific COD techniques may be beneficial.
